# Exposure to Head Impacts and Cognitive and Behavioral Outcomes in Youth Tackle Football Players Across 4 Seasons

**DOI:** 10.1001/jamanetworkopen.2021.40359

**Published:** 2021-12-30

**Authors:** Sean C. Rose, Keith Owen Yeates, Joseph T. Nguyen, Natalie M. Pizzimenti, Patrick M. Ercole, Matthew T. McCarthy

**Affiliations:** 1 Child Neurology, Nationwide Children’s Hospital, The Ohio State University, Columbus; 2 Department of Psychology, Alberta Children’s Hospital Research Institute, Hotchkiss Brain Institute, University of Calgary, Calgary, Alberta, Canada; 3 Sansom Consulting, San Antonio, Texas; 4 MORE Foundation, Phoenix, Arizona; 5 Summit Health, Florham Park, New Jersey

## Abstract

**Importance:**

Repetitive head impacts have been posited to contribute to neurocognitive and behavioral difficulties in contact sport athletes.

**Objective:**

To identify associations between cognitive and behavioral outcomes and head impacts measured in youth tackle football players over 4 seasons of play.

**Design, Setting, and Participants:**

This prospective cohort study was conducted from July 2016 through January 2020, spanning 4 football seasons. The setting was a youth tackle football program and outpatient medical clinic. Players were recruited from 4 football teams composed of fifth and sixth graders, and all interested players who volunteered to participate were enrolled. Data analysis was performed from March 2020 to June 2021.

**Exposures:**

Impacts were measured using helmet-based sensors during practices and games throughout 4 consecutive seasons of play. Impacts were summed to yield cumulative head impact gravitational force equivalents per season.

**Main Outcomes and Measures:**

Ten cognitive and behavioral measures were completed before and after each football season.

**Results:**

There were 70 male participants aged 9 to 12 years (mean [SD] age, 10.6 [0.64] years), with 18 completing all 4 years of the study. At the post–season 1 time point, higher cumulative impacts were associated with lower self-reported symptom burden (β = −0.6; 95% CI, −1.0 to −0.2; *P* = .004). After correcting for multiple comparisons, no other associations were found between impacts and outcome measures. At multiple times throughout the study, premorbid attention-deficit/hyperactivity disorder, anxiety, and depression were associated with worse cognitive or behavioral scores, whereas a premorbid headache disorder or history of concussion was less often associated with outcomes.

**Conclusions and Relevance:**

In this cohort of youth tackle football players, premorbid conditions, including attention-deficit/hyperactivity disorder, anxiety, and depression, were associated with cognitive and behavioral outcomes more often than cumulative impact.

## Key Points

**Question:**

Are repetitive head impacts in youth tackle football across 4 years of play associated with cognitive and behavioral difficulties?

**Findings:**

In this cohort study including 70 male participants aged 9 to 12 years, few associations were found between cumulative head impacts and cognitive and behavioral outcomes, and no associations were seen consistently or became more prominent over the years.

**Meaning:**

Because head impacts were not associated with measured outcomes, these findings suggest that other factors, including premorbid medical conditions, should be further explored.

## Teaser

This cohort study examines the association between cognitive and behavioral outcomes and head impacts measured in youth tackle football players over 4 seasons of play.

## Introduction

Repetitive subconcussive head impacts during childhood have been implicated in the development of chronic cognitive and behavioral problems. However, both retrospective^1,2,3,4,5,6,7^ and prospective^8,9,10,11,12^ research studies have yielded conflicting results regarding the association between repetitive head impacts and cognitive and behavioral outcomes.

Previous studies^8,9,10,13^ in pre–high school athletes have generally examined 1 year of head impact exposure. We conducted a 4-year prospective study in youth tackle football players. We previously reported few associations between measured head impacts and neurobehavioral outcomes over the course of 3 seasons of play.^11^ The current study sought to identify associations between cognitive and behavioral outcomes and head impacts measured in youth tackle football players over 4 seasons of play.

## Methods

### Participants

This cohort study was approved by the IntegReview institutional review board. Written consent and assent were obtained from a parent and all participants, respectively. This study followed the Strengthening the Reporting of Observational Studies in Epidemiology (STROBE) reporting guideline.

A local youth tackle football program was identified through community engagement; the program leadership and coaches expressed interest in participating in research examining the safety of youth tackle football. The fifth and sixth grade teams were selected for this study at the recommendation of the coaches to prioritize young age but also large team size. Players entering the fifth and sixth grades were enrolled in the summer of 2016 and followed through the fall football seasons of 2016 (season 1), 2017 (season 2), 2018 (season 3), and 2019 (season 4). Players dropped out of the study if they stopped playing football or if they did not attend preseason or postseason testing visits.

### Impact Monitoring

Head impacts were monitored using helmet-based Riddell InSite sensors during practices and games. InSite sensors were first developed to quantify blunt force trauma and overpressurization from military blast injuries.^14^ They characterize linear acceleration using ferroelectret films that produce an electrical charge that is proportional to the deformation of the sensor during an impact event. The relationship between sensor deformation and head acceleration is based on direct comparison testing showing a strong correlation between InSite sensors and accelerometers embedded in Hybrid III head forms (*r*^2^, 0.900-0.963).^15^ In the current study, a head impact was defined as any impact detected by the InSite sensor. During season 1, the sensors detected impacts greater than or equal to 10 gravitational force equivalents (*g*). As a result of a change in the manufacturer’s software to account for changes in the manufacturing process of the ferroelectret film, during seasons 2 to 4 the sensors detected impacts greater than or equal to 15*g*. The cumulative impact for each player for each season was calculated according to previously reported methods.^11,12^

### Neurocognitive and Behavioral Assessments

Before and after each football season, players completed several cognitive and behavioral assessments (Table 1). The Medical Symptom Validity Test was used at each visit to screen for response validity.

**Table 1.** Neurocognitive and Behavioral Tests Used as Outcomes Measures

Type of tests	Description of assessment
Cognitive tests	
Wechsler Abbreviated Scale of Intelligence Full-Scale Intelligence Quotient 2 subtests	Vocabulary subtest; word knowledge
	Matrix reasoning subtest; nonverbal reasoning and problem solving
Wechsler Intelligence Scale for Children V coding subtest	Processing speed
Wechsler Intelligence Scale for Children V digit span subtest	Working memory
Child and Adolescent Memory Profile Index Score	Lists subtest; verbal memory
	Objects subtest; visual memory
Trail-Making Test Condition 4	Visual attention and task-switching
Test of Variables of Attention Response Time Variability	Attention and inhibitory control
CogState processing speed subtest	Brief computerized test of processing speed
Behavioral and symptom questionnaires	
Strengths and Weaknesses of ADHD Symptoms and Normal Behavior Rating Scale	Parent report of ADHD-related symptoms of inattention and hyperactivity
Strengths and Difficulties Questionnaire	Total difficulties subsection: self-reported difficulties with mood, behavior, and social adjustment
Sport Concussion Assessment Tool–3	Self-report of common concussion symptoms

Abbreviation: ADHD, attention-deficit/hyperactivity disorder.

Previous medical diagnoses, including headaches, migraines, attention-deficit/hyperactivity disorder (ADHD), anxiety, depression, and number of prior concussions, were recorded at the pre–season 1 visit. Interval concussions were documented at follow-up visits. At the final post–season 4 visit, players reported whether they played other contact sports (defined as wrestling, ice hockey, soccer, lacrosse, or rugby).

### Statistical Analysis

Three players were excluded from statistical analyses at the corresponding assessment time point because of Medical Symptom Validity Test failure (2 players before season 1 and 1 player after season 1). Because of missing impact data, additional players were excluded from statistical analyses involving the corresponding season’s cumulative impact (9 players in season 1 and 3 players in season 4).

In previous reports,^9,11,12^ we found several outcome measures suggesting a potential association with cumulative impact. On the basis of these results and associations found in other studies,^8,16^ we narrowed the list of outcome measures in the current analysis to the 10 most likely to be affected by repetitive head impacts (Table 1).

To examine the potential for attrition bias, independent-samples 2-sided *t* tests and χ^2^ tests were used to compare the players who were included in the final post–season 4 analysis and the players who contributed data to fewer time points. Linear mixed models were used to examine change in outcome measures over time. Linear mixed models with fixed-effect factors of premorbid medical diagnoses assessed whether outcome trends varied by diagnoses over time. The Wald χ^2^ statistic was used to determine the effect of time as a discrete measure and then analyzed as a series of pairwise comparisons among the estimated marginal means. Multivariable linear regressions were used to determine the association of cumulative head impact with each outcome measure, controlling for premorbid medical diagnoses. Each analysis accounted for the changing sample size over time and the cumulative nature of the head impact data with each subsequent season. These models included all head impacts measured prior to any given time point.

Significance was defined as α < .05. All statistical analyses were performed using SPSS statistical software for Mac version 24.0 (IBM Corporation) and R statistical software version 4.0.3 (R Project for Statistical Computing). Data analysis was performed from March 2020 to June 2021.

## Results

Seventy male players aged 9 to 12 years (mean [SD] age, 10.6 [0.64] years) from a single youth tackle football program enrolled and completed the pre–season 1 assessment; 18 players completed all 4 years of the study. Age, mean cumulative impact per season, and preenrollment medical diagnoses are shown in Table 2. During each of the 4 seasons, 1 player received a diagnosis of concussion, with the same player sustaining a concussion in seasons 2 and 3. For each diagnosed concussion, symptoms had resolved and the player was medically cleared before completing their postseason assessment. Premorbid diagnoses, pre–season 1 outcome measures, and cumulative impacts in season 1 did not differ between players who completed all 4 years of the study and those who dropped out (eTable 1 in the Supplement).

**Table 2.** Age, Premorbid Diagnoses, and Mean Cumulative Impact

Variable	Participants, No. (%)
Age at first season, y	
Mean (SD), y	10.6 (0.64)
9	4 (6)
10	23 (33)
11	41 (59)
12	2 (3)
Headaches or migraines	
No	42 (60)
Yes	28 (40)
Attention-deficit/hyperactivity disorder	
No	61 (87)
Yes	9 (13)
Anxiety or depression	
No	65 (93)
Yes	5 (7)
Any prior concussions	
No	62 (89)
Yes	8 (11)
Prior concussions, No.	
0	62 (89)
1	6 (9)
2	2 (3)
Cumulative impact per season, mean (SD), *g*	
Season 1^a^	4117 (3254)
Season 2	2841 (1976)
Season 3	5817 (4541)
Season 4	2991 (2018)

Abbreviation: *g*, gravitational force equivalent.

^a^ Season 1 recorded impacts greater than 10*g*, whereas seasons 2 through 4 recorded impacts greater than 15*g*.

### Change in Outcome Scores Over Time, Independent of Cumulative Impacts

Most outcome measures improved over time except for Test of Variables of Attention Response Time Variability (Wald χ^2^_7_ = 4.86; *P* = .68) and Sport Concussion Assessment Tool–3 Total (Wald χ^2^_7_ = 6.08; *P* = .53), which did not change over time. The Strengths and Weaknesses of ADHD Symptoms and Normal Behavior Rating Scale Total fluctuated over time (Wald χ^2^_7_ = 15.57; *P* = .03). The CogState Processing Speed worsened over time (Wald χ^2^_7_ = 79.02; *P* < .001).

### Associations Between Cumulative Impact Over Time and Outcome Scores

When assessing the association of cumulative impact measured in all previous years up to each assessment time point, few associations were significant (Table 3). Higher cumulative impact was associated with lower Sport Concussion Assessment Tool–3 symptom scores after season 1 and before season 2, but higher scores before season 3. At the pre–season 3 time point, higher cumulative impact was also associated with worse concentration on Test of Variables of Attention. Once a Bonferroni correction was applied (*P* < .05/10 or .005), only the association with the post–season 1 Sport Concussion Assessment Tool–3 score remained significant (β = −0.6; 95% CI, −1.0 to −0.2; *P* = .004). When playing other contact sports was added to the model as a covariate, a few specific associations were identified only at pre–season 2, but no other statistically significant associations were detected between cumulative impact and outcome measures or between playing other contact sports and outcome measures.

**Table 3.** Association of Cumulative Impacts With Neurocognitive and Behavioral Outcomes at Each Time Point

Season and measure	β (95% CI)^a^	*P* value
Season 1 (postseason) (n = 55)		
WASI FSIQ 2	0.2 (−0.8 to 1.2)	.66
WISC		
Digits	−0.1 (−0.3 to 0.2)	.62
Coding	−0.1 (−0.4 to 0.2)	.65
ChAMP Index score	−0.3 (−1.6 to 1.0)	.67
TMT condition 4	0.0 (−0.2 to 0.2)	.90
TOVA VARZT	−0.1 (−0.3 to 0.1)	.17
CogState Processing Speed	0.8 (−0.2 to 1.7)	.12
SWAN total	0.1 (−0.2 to 0.4)	.49
SDQ total difficulties	0.0 (−0.4 to 0.3)	.78
SCAT-3 total	−0.6 (−1.0 to −0.2)	.004^b^
Season 2		
Preseason (n = 41)		
WASI FSIQ 2	−0.7 (−2.1 to 0.6)	.27
WISC		
Digits	0.1 (−0.2 to 0.4)	.41
Coding	−0.1 (−0.5 to 0.2)	.53
ChAMP Index score	0.7 (−0.5 to 1.9)	.24
TMT condition 4	0.0 (−0.3 to 0.3)	.92
TOVA VARZT	0.0 (−0.2 to 0.2)	.74
CogState Processing Speed	−1.1 (−2.8 to 0.6)	.21
SWAN total	−0.1 (−0.4 to 0.2)	.47
SDQ total difficulties	0.0 (−0.4 to 0.5)	.92
SCAT-3 total	−0.4 (−0.7 to 0.0)	.04^c^
Postseason (n = 36)		
WASI FSIQ 2	0.3 (−0.6 to 1.3)	.46
WISC		
Digits	0.1 (−0.1 to 0.4)	.34
Coding	0.0 (−0.2 to 0.3	.74
ChAMP Index score	0.5 (−0.4 to 1.5)	.27
TMT condition 4	0.0 (−0.3 to 0.2)	.79
TOVA VARZT	0.1 (−0.1 to 0.2)	.39
CogState Processing Speed	0.4 (−0.5 to 1.2)	.42
SWAN total	0.1 (−0.2 to 0.3)	.62
SDQ total difficulties	0.0 (−0.2 to 0.3)	.84
SCAT-3 total	0.2 (−0.2 to 0.6)	.37
Season 3		
Preseason (n = 31)		
WASI FSIQ 2	0.6 (−0.5 to 1.6)	.25
WISC		
Digits	0.0 (−0.2 to 0.2)	.93
Coding	0.1 (−0.2 to 0.3)	.63
ChAMP Index score	0.4 (−0.7 to 1.5)	.45
TMT Condition 4	0.0 (−0.1 to 0.2)	.62
TOVA VARZT	−0.2 (−0.4 to 0.0)	.03^c^
CogState Processing Speed	−0.1 (−1.0 to 0.7)	.73
SWAN total	0.0 (−0.3 to 0.3)	.78
SDQ total difficulties	0.2 (−0.1 to 0.4)	.26
SCAT-3 total	0.4 (0.1 to 0.7)	.02^c^
Postseason (n = 29)		
WASI FSIQ 2	0.1 (−0.7 to 0.8)	.87
WISC		
Digits	−0.1 (−0.2 to 0.1)	.40
Coding	0.0 (−0.2 to 0.2)	.77
ChAMP Index score	0.2 (−0.5 to 0.8)	.65
TMT Condition 4	0.0 (−0.1 to 0.1)	.77
TOVA VARZT	−0.1 (−0.1 to 0.0)	.14
CogState Processing Speed	0.0 (−0.8 to 0.8)	.98
SWAN total	0.0 (−0.1 to 0.1)	.72
SDQ total difficulties	0.0 (−0.2 to 0.2)	.94
SCAT-3 total	0.1 (−0.1 to 0.3)	.52
Season 4		
Preseason (n = 24)		
WASI FSIQ 2	0.4 (−0.6 to 1.4)	.45
WISC		
Digits	0.0 (−0.2 to 0.2)	.80
Coding	−0.1 (−0.3 to 0.1)	.48
ChAMP Index score	0.3 (−0.6 to 1.1)	.49
TMT condition 4	0.1 (−0.1 to 0.2)	.43
TOVA VARZT	0.0 (−0.1 to 0.1)	.88
CogState Processing Speed	0.0 (−1.1 to 1.0)	.95
SWAN total	0.0 (−0.1 to 0.1)	.73
SDQ Total difficulties	−0.1 (−0.3 to 0.1)	.22
SCAT-3 total	−0.2 (−0.4 to 0.1)	.16
Postseason (n = 18)		
WASI FSIQ 2	0.4 (−0.6 to 1.4)	.40
WISC		
Digits	0.0 (−0.2 to 0.3)	.70
Coding	0.0 (−0.3 to 0.2)	.69
ChAMP Index score	0.2 (−0.4 to 0.8)	.52
TMT condition 4	0.0 (−0.1 to 0.1)	.93
TOVA VARZT	0.0 (−0.2 to 0.1)	.39
CogState Processing Speed	0.0 (−1.0 to 1.0)	.96
SWAN total	0.0 (−0.1 to 0.2)	.78
SDQ total difficulties	0.0 (−0.2 to 0.3)	.92
SCAT-3 total	0.0 (−0.2 to 0.2)	.81

Abbreviations: ChAMP, Child and Adolescent Memory Profile; SCAT-3, Sport Concussion Assessment Tool 3rd Edition symptom score; SDQ, Strengths and Difficulties Questionnaire; SWAN, Strengths and Weakness of ADHD Symptoms and Normal Behavior Rating Scale; TMT, Trail Making Test; TOVA, Test of Variables of Attention; VARZT, Response Time Variability *z* score; WASI FSIQ 2, Wechsler Abbreviated Scale of Intelligence 2nd Edition Full Scale Intelligence Quotient 2 subtests; WISC, Wechsler Intelligence Scale for Children 5th Edition.

^a^ Multivariate linear regressions examined the association of cumulative impact with outcome measures. β indicates the direction of affect, with a more positive value indicating an increase in score and a more negative value indicating a decrease. Increase is considered an improvement for all measures except for SWAN Total, SDQ Total Difficulties, and SCAT-3 Total.

^b^ Statistically significant after Bonferroni correction (0.05/10 = *P* < .005).

^c^ Statistically significant at *P* < .05.

### Association Between Premorbid Medical Conditions and Outcome Scores

Players with premorbid medical conditions performed worse on several outcome measures during the study period (eTable 2 in the Supplement). For example, players with a history of ADHD performed worse on Wechsler Abbreviated Scale of Intelligence Full-Scale Intelligence Quotient–2, Wechsler Intelligence Scale for Children 5th Edition Digits, Test of Variables of Attention Response Time Variability, CogState Processing Speed, Strengths and Weaknesses of ADHD Symptoms and Normal Behavior Rating Scale Total, and Strengths and Difficulties Questionnaire Total Difficulties, with the between-group difference often becoming more prominent as the study progressed. Players with a history of anxiety or depression had worse scores on the Wechsler Abbreviated Scale of Intelligence Full-Scale Intelligence Quotient–2 or Strengths and Difficulties Questionnaire Total Difficulties at 6 of 8 and 5 of 8 time points, respectively.

## Discussion

Some previous retrospective studies^3,4^ have suggested that exposure to repetitive head impacts in tackle football before the age of 12 years is associated with neurobehavioral and cognitive problems later in life. However, other studies^1,2,6,7^ have found no association between earlier age of exposure to tackle football or other contact sports and deleterious outcomes. Additionally, current high school and collegiate athletes with head impact exposure before age 12 years have not been shown to have worse neurocognitive performance than those with exposure after age 12 years.^17^

Here we contribute evidence from children who were aged 9 to 12 years at enrollment that repetitive head impacts in youth tackle football were not found to be associated with neurocognitive performance or behavioral outcomes. Although outcomes on a computerized measure of processing speed (CogState) declined over the course of this study, a well-validated measure of processing speed (Wechsler Intelligence Scale for Children 5th Edition coding) improved over time, and neither change was associated with head impacts. To our knowledge, this is the longest prospective study to date to measure head impacts and neurocognitive outcomes in youth contact sport athletes. Consistent with the first 3 years of this study, our findings from 4 seasons of play did not identify an association between cumulative head impact and neurocognitive outcomes.

### Limitations

This study has limitations that should be considered. First, premorbid medical diagnoses were reported by the player and a parent, but formal diagnostic criteria were not verified. Second, the attrition of players over time limited the statistical power in the final years of the study. To reduce the effect of the decreasing sample size over time and to reduce the likelihood of type I error associated with multiple comparisons, we analyzed only the 10 outcome measures conceptually associated with repetitive head impacts. In addition, players who dropped out of the study did not differ from those who continued through all 4 years. Third, all impact sensors have limitations, and we have previously described the characteristics of the Riddell InSite sensors.^9,11,12^ We did not confirm each helmet impact with video recordings of practices and games. Fourth, although this was a prospective study spanning 4 years, we were unable to determine long-term neurocognitive outcomes in our participants. Future prospective studies could measure head impacts and monitor outcomes throughout life.

## Conclusions

In conclusion, we did not find compelling evidence that cumulative head impacts across 4 years of play are associated with neurocognitive function in youth tackle football players. Rather, self-reported medical diagnoses, especially ADHD, anxiety, and depression, were consistently associated with worse neurocognitive outcomes. Over time, neurocognitive performance appears to be influenced by comorbid medical diagnoses more than by repetitive head impacts.

## Notes

Supplement. **eTable 1.** Comparison of Characteristics at the Preseason 1 Assessment Between Players With Incomplete Data and Players With Complete Data **eTable 2.** Premorbid Medical Conditions and Outcome Measures Over Time

## References

[1] Caccese J, Iverson GL, Cameron K, . Estimated age of first exposure to contact sports is not associated with greater symptoms or worse cognitive functioning in U.S. Service Academy athletes. J Neurotrauma. 2020;37(2):334-339. doi:10.1089/neu.2019.6571 31375052 PMC7364303

[2] Caccese JB, DeWolf RM, Kaminski TW, ; CARE Consortium Investigators. Estimated age of first exposure to American football and neurocognitive performance amongst NCAA male student-athletes: a cohort study. Sports Med. 2019;49(3):477-487. doi:10.1007/s40279-019-01069-x 30747378

[3] Stamm JM, Bourlas AP, Baugh CM, . Age of first exposure to football and later-life cognitive impairment in former NFL players. Neurology. 2015;84(11):1114-1120. doi:10.1212/WNL.0000000000001358 25632088 PMC4371403

[4] Alosco ML, Kasimis AB, Stamm JM, . Age of first exposure to American football and long-term neuropsychiatric and cognitive outcomes. Transl Psychiatry. 2017;7(9):e1236. doi:10.1038/tp.2017.197 28926003 PMC5639242

[5] Deshpande SK, Hasegawa RB, Rabinowitz AR, . Association of playing high school football with cognition and mental health later in life. JAMA Neurol. 2017;74(8):909-918. doi:10.1001/jamaneurol.2017.1317 28672325 PMC5710329

[6] Solomon GS, Kuhn AW, Zuckerman SL, . Participation in pre-high school football and neurological, neuroradiological, and neuropsychological findings in later life: a study of 45 retired National Football League players. Am J Sports Med. 2016;44(5):1106-1115. doi:10.1177/0363546515626164 26888877

[7] Iverson GL, Caccese JB, Merz ZC, Büttner F, Terry DP. Age of first exposure to football is not associated with later-in-life cognitive or mental health problems. Front Neurol. 2021;12:647314. doi:10.3389/fneur.2021.647314 34025554 PMC8131846

[8] Maerlender A, Smith E, Brolinson PG, . Neuropsychological change after a single season of head impact exposure in youth football. J Int Neuropsychol Soc. 2021;27(2):113-123. doi:10.1017/S1355617720000685 32762785 PMC7867662

[9] Rose SC, Yeates KO, Fuerst DR, Ercole PM, Nguyen JT, Pizzimenti NM. Head impact burden and change in neurocognitive function during a season of youth football. J Head Trauma Rehabil. 2019;34(2):87-95. doi:10.1097/HTR.0000000000000441 30320727

[10] Munce TA, Dorman JC, Thompson PA, Valentine VD, Bergeron MF. Head impact exposure and neurologic function of youth football players. Med Sci Sports Exerc. 2015;47(8):1567-1576. doi:10.1249/MSS.0000000000000591 25437194

[11] Rose SC, Yeates KO, Nguyen JT, Ercole PM, Pizzimenti NM, McCarthy MT. Subconcussive head impacts and neurocognitive function over 3 seasons of youth football. J Child Neurol. 2021;36(9):768-775. doi:10.1177/08830738211004490 33834862

[12] Rose SC, Yeates KO, Nguyen JT, McCarthy MT, Ercole PM, Pizzimenti NM. Neurocognitive function and head impact burden over two seasons of youth tackle football. J Neurotrauma. 2019;36(19):2803-2809. doi:10.1089/neu.2019.6519 31084394

[13] Jennings D, Sells P, Allison J, . Effects of a season of subconcussive contact on child-SCAT3 scores in 8-12 year-old male athletes. Int J Sports Phys Ther. 2015;10(5):667-675. 26491617 PMC4595920

[14] Chu JJ, Beckwith JG, Leonard DS, Paye CM, Greenwald RM. Development of a multimodal blast sensor for measurement of head impact and over-pressurization exposure. Ann Biomed Eng. 2012;40(1):203-212. doi:10.1007/s10439-011-0410-6 21994064

[15] Beckwith JG, Chu JJ, Leonard DS, Bolander RP, Buck AT, Greenwald RM. Evaluation of a low-cost thin film sensor for head impact exposure monitoring. 38th Annual Meeting of the American Society of Biomechanics and World Congress of Biomechanics. July 2014. Accessed November 29, 2021. https://www.asbweb.org/wp-content/uploads/2014WCBprogramandother.pdf

[16] McAllister TW, Flashman LA, Maerlender A, . Cognitive effects of one season of head impacts in a cohort of collegiate contact sport athletes. Neurology. 2012;78(22):1777-1784. doi:10.1212/WNL.0b013e3182582fe7 22592370 PMC3359587

[17] Brett BL, Huber DL, Wild A, Nelson LD, McCrea MA. Age of first exposure to American football and behavioral, cognitive, psychological, and physical outcomes in high school and collegiate football players. Sports Health. 2019;11(4):332-342. doi:10.1177/1941738119849076 31173699 PMC6600580

