# Supplementary material for: Exposure to Head Impacts and Cognitive and Behavioral Outcomes in Youth Tackle Football Players Across 4 Seasons
Source: JAMA Netw Open. 2021 Dec 30;4(12):e2140359. doi: 10.1001/jamanetworkopen.2021.40359 (PMC8719231; doi:10.1001/jamanetworkopen.2021.40359)
Supplement: Supplement. — eTable 1. Comparison of Characteristics at the Preseason 1 Assessment Between Players With Incomplete Data and Players With Complete Data eTable 2. Premorbid Medical Conditions and Outcome Measures Over Time [file jamanetwopen-e2140359-s001.pdf]

Supplemental Online Content

Rose SC, Yeates KO, Nguyen JT, Pizzimenti NM, Ercole PM, McCarthy MT. Exposure to head impacts and cognitive and behavioral outcomes in youth tackle football players across 4 seasons. *JAMA Netw Open*. 2021;4(12):e2140359. doi:10.1001/jamanetworkopen.2021.40359

eTable 1. Comparison of Characteristics at the Preseason 1 Assessment Between Players With Incomplete Data and Players With Complete Data

eTable 2. Premorbid Medical Conditions and Outcome Measures Over Time

This supplemental material has been provided by the authors to give readers additional information about their work.

© 2021 Rose SC et al. *JAMA Network Open.*

eTable 1. Comparison of Characteristics at the Preseason 1 Assessment Between Players With Incomplete Data and Players With Complete Data

Variable	Group	Dropped out (N=52)		All 4 seasons (N=18)		F-statistic or Chi-square statistic	P-value
		Mean	SD	Mean	SD		
**Age at first season**		10.5	0.7	10.9	0.4	8.20	0.006*
**Cumulative Impact in season 1**		3.8	3.3	4.8	3.3	1.00	0.32
**WASI FSIQ 2**		103.3	9.9	99.7	12.8	1.50	0.23
**WISC Digits**		10.7	2.7	10.8	3.1	0.04	0.84
**WISC Coding**		8.4	2.7	9.5	2.3	2.44	0.12
**ChAMP Index Score**		103.1	11.7	107.3	10.4	1.82	0.18
**TMT Condition 4**		10.1	2.4	9.0	3.7	2.17	0.15
**TOVA VARZT**		-2.4	2.3	-2.6	2.5	0.13	0.72
**CogState Processing Speed**		103.8	10.4	104.4	11.4	0.04	0.84
**SWAN Total**		2.4	3.3	2.5	2.8	0.02	0.89
**SDQ Total Difficulties**		6.6	3.8	6.2	4.2	0.14	0.71
**SCAT-3 Total**		3.0	3.7	2.3	3.6	0.57	0.46
**Headaches or Migraines**	No	28	54%	14	78%	3.19	0.07
	Yes	24	46%	4	22%		
**ADHD**	No	45	87%	16	89%	0.07	0.80
	Yes	7	13%	2	11%		
**Anxiety or Depression**	No	49	94%	16	89%	0.58	0.45
	Yes	3	6%	2	11%		
**Any prior concussions**	No	48	92%	14	78%	2.79	0.10
	Yes	4	8%	4	22%		

WASI FSIQ 2: Wechsler Abbreviated Scale of Intelligence 2nd Edition Full Scale intelligence quotient two subtests, WISC: Wechsler Intelligence Scale for Children 5th Edition, ChAMP: Child and Adolescent Memory Profile, TMT: Trail Making Test, TOVA: Test of Variables of Attention, VARZT: Response Time Variability z-score, SWAN: Strengths and Weakness of ADHD Symptoms and Normal Behavior Rating Scale, SDQ: Strengths and Difficulties Questionnaire, SCAT-3: Sport Concussion Assessment Tool 3rd Edition symptom score, ADHD: attention deficit hyperactivity disorder. Degrees of freedom = 1 for all tests. *Statistically significant at $p<0.05$.

© 2021 Rose SC et al. *JAMA Network Open.*

eTable 2. Premorbid Medical Donditions and Outcome Measures Over Time

		ADHD					Anxiety/Depression					Headache/Migraine					Previous Concussion(s)				
		No		Yes		P-value	No		Yes		P-value	No		Yes		P-value	No		Yes		P-value
		Mean	SE	Mean	SE		Mean	SE	Mean	SE		Mean	SE	Mean	SE		Mean	SE	Mean	SE	
**WASI FSIQ 2**	Season 1 pre	103.1	1.4	97.4	3.2	0.101	102.7	1.4	98.4	2.4	0.120	100.4	1.7	105.4	1.9	0.044	102.2	1.4	103.7	3.8	0.715
	Season 1 post	104.0	1.5	96.2	2.7	0.012	103.7	1.4	94.4	2.6	0.002	103.3	1.7	102.5	2.2	0.794	102.4	1.5	106.8	3.7	0.283
	Season 2 pre	106.1	1.9	101.3	5.5	0.410	106.7	1.9	92.3	2.8	0.000	107.6	2.2	102.2	3.1	0.151	104.8	1.7	110.3	7.6	0.474
	Season 2 post	107.4	1.9	97.0	4.5	0.033	107.4	1.8	95.0	6.5	0.067	109.2	2.1	100.6	2.9	0.017	105.9	2.0	107.8	4.3	0.682
	Season 3 pre	106.1	2.1	94.8	5.7	0.063	106.3	2.1	93.3	3.4	0.001	107.2	2.0	100.3	4.2	0.131	104.3	2.2	107.2	4.8	0.590
	Season 3 post	109.3	2.2	95.5	4.6	0.007	108.6	2.3	98.3	2.7	0.004	108.7	2.4	105.2	4.2	0.475	107.1	2.2	110.2	6.7	0.663
	Season 4 pre	106.5	2.9	92.7	4.7	0.013	106.8	2.8	90.3	5.2	0.005	107.8	2.8	99.2	5.7	0.172	105.4	2.9	102.3	8.2	0.713
	Season 4 post	110.6	3.1	101.5	1.1	0.005	110.9	3.0	98.0	3.5	0.005	109.9	3.4	109.6	5.2	0.961	110.3	2.8	107.5	9.3	0.777
**WISC Digits**	Season 1 pre	10.8	0.4	10.4	0.8	0.713	10.8	0.4	10.2	0.7	0.451	10.5	0.4	11.0	0.5	0.440	10.7	0.4	10.9	0.9	0.874
	Season 1 post	10.8	0.4	9.9	1.0	0.369	10.8	0.3	9.8	1.3	0.475	10.4	0.4	11.1	0.6	0.374	10.6	0.4	11.4	0.5	0.195
	Season 2 pre	10.7	0.4	9.3	0.7	0.091	10.6	0.4	10.5	0.4	0.918	10.7	0.5	10.3	0.6	0.583	10.6	0.4	10.0	0.5	0.339
	Season 2 post	11.6	0.5	10.0	0.8	0.095	11.5	0.5	11.0	0.6	0.541	11.4	0.5	11.6	0.9	0.839	11.6	0.5	10.2	0.9	0.173
	Season 3 pre	11.4	0.5	8.5	0.8	0.002	11.1	0.5	10.3	1.1	0.456	11.5	0.5	10.3	0.9	0.230	11.1	0.5	10.4	0.9	0.480
	Season 3 post	11.5	0.5	9.3	1.1	0.065	11.3	0.5	11.0	0.9	0.797	11.4	0.5	10.9	0.9	0.637	11.5	0.5	10.0	0.9	0.152
	Season 4 pre	12.1	0.6	10.3	1.4	0.249	12.0	0.6	11.3	0.7	0.482	12.3	0.6	11.1	1.0	0.305	12.1	0.6	11.0	0.9	0.334
	Season 4 post	12.5	0.7	10.5	0.4	0.012	12.4	0.7	11.5	0.4	0.281	12.0	0.9	13.0	0.5	0.318	12.6	0.7	10.8	1.0	0.123
**WISC Coding**	Season 1 pre	8.7	0.3	8.2	1.1	0.659	8.7	0.3	8.0	1.1	0.535	8.5	0.4	8.9	0.5	0.594	8.5	0.3	9.9	0.8	0.147
	Season 1 post	9.8	0.4	9.7	1.1	0.907	9.8	0.4	10.2	1.0	0.659	9.7	0.4	10.0	0.6	0.675	9.8	0.4	10.0	0.7	0.747
	Season 2 pre	10.1	0.5	9.8	1.3	0.830	10.0	0.5	11.3	0.8	0.176	10.8	0.6	9.1	0.6	0.037	9.8	0.5	11.8	1.1	0.083
	Season 2 post	11.5	0.5	11.6	1.6	0.939	11.3	0.5	13.0	0.9	0.110	11.3	0.6	11.8	0.7	0.610	11.3	0.5	13.0	1.2	0.169
	Season 3 pre	10.4	0.5	10.5	2.1	0.951	10.3	0.5	11.3	0.5	0.201	10.5	0.6	10.1	0.8	0.646	10.1	0.5	12.0	0.4	0.005
	Season 3 post	10.8	0.6	10.3	1.2	0.688	10.8	0.6	10.3	0.7	0.649	10.5	0.7	11.1	0.9	0.622	10.6	0.6	11.6	1.2	0.433
	Season 4 pre	11.9	0.6	12.7	1.2	0.570	12.0	0.6	12.0	0.8	1.000	11.9	0.7	12.1	0.6	0.862	11.9	0.6	12.8	1.6	0.610
	Season 4 post	13.2	0.8	15.0	1.4	0.253	13.5	0.8	11.5	1.1	0.130	13.1	0.9	14.0	1.0	0.492	13.5	0.8	12.5	1.5	0.563

© 2021 Rose SC et al. *JAMA Network Open.*

		ADHD					Anxiety/Depression					Headache/Migraine					Previous Concussion(s)				
		No		Yes		P-value	No		Yes		P-value	No		Yes		P-value	No		Yes		P-value
		Mean	SE	Mean	SE		Mean	SE	Mean	SE		Mean	SE	Mean	SE		Mean	SE	Mean	SE	
**ChAMP Index Score**	Season 1 pre	104.3	1.3	103.2	5.7	0.850	103.8	1.4	109.2	4.8	0.278	102.4	1.6	106.9	2.4	0.121	104.0	1.5	105.9	2.5	0.521
	Season 1 post	113.0	1.5	107.3	6.9	0.419	111.9	1.7	116.2	3.2	0.236	111.8	1.4	112.9	3.4	0.766	111.7	1.8	116.3	2.1	0.101
	Season 2 pre	116.4	1.6	109.0	5.5	0.198	115.0	1.7	119.8	5.7	0.422	117.2	1.9	112.6	2.8	0.165	114.5	1.8	121.5	2.6	0.026
	Season 2 post	121.6	1.8	112.6	8.1	0.277	120.1	2.1	122.8	4.3	0.581	123.3	2.2	115.4	3.3	0.045	119.6	2.2	125.4	2.1	0.058
	Season 3 pre	119.4	2.1	108.8	10.5	0.320	117.6	2.5	121.8	4.6	0.436	122.4	1.9	110.3	4.8	0.018	117.5	2.6	121.6	4.8	0.454
	Season 3 post	122.2	2.3	121.8	2.9	0.908	121.2	2.2	131.0	2.2	0.001	124.3	2.1	117.3	4.3	0.144	121.2	2.3	127.2	2.3	0.070
	Season 4 pre	126.3	1.4	109.7	17.0	0.330	123.7	2.7	130.7	3.3	0.105	126.9	1.6	119.4	6.4	0.257	123.8	2.8	128.0	4.2	0.405
	Season 4 post	123.8	2.0	121.5	1.1	0.310	124.2	1.9	117.0	2.1	0.012	124.4	2.0	121.7	3.5	0.512	123.7	2.0	123.0	3.8	0.874
**TMT Condition 4**	Season 1 pre	9.8	0.4	10.2	0.5	0.492	10.0	0.4	8.0	0.8	0.031	9.6	0.5	10.2	0.5	0.392	10.0	0.4	8.6	0.5	0.029
	Season 1 post	10.9	0.3	10.0	0.8	0.264	10.8	0.3	11.2	0.9	0.668	10.8	0.3	10.8	0.5	0.915	10.8	0.3	11.1	0.5	0.567
	Season 2 pre	10.7	0.4	10.0	1.2	0.563	10.8	0.4	9.0	2.3	0.447	10.8	0.4	10.4	0.8	0.657	10.5	0.4	11.3	0.8	0.394
	Season 2 post	10.7	0.5	9.2	1.7	0.382	10.4	0.5	12.0	0.4	0.012	10.7	0.6	10.2	0.9	0.631	10.3	0.6	12.2	0.4	0.007
	Season 3 pre	11.3	0.4	11.0	0.8	0.731	11.4	0.4	10.0	0.5	0.021	11.4	0.4	11.1	0.6	0.704	11.4	0.4	10.6	0.5	0.193
	Season 3 post	11.7	0.3	12.5	0.3	0.052	11.8	0.3	12.0	0.0	0.510	11.6	0.3	12.3	0.6	0.273	11.9	0.3	11.2	0.5	0.235
	Season 4 pre	12.1	0.4	11.0	0.8	0.226	12.1	0.4	10.7	0.5	0.027	12.0	0.5	11.9	0.5	0.867	12.0	0.4	11.5	1.0	0.619
	Season 4 post	12.2	0.5	12.5	0.4	0.594	12.3	0.5	11.5	0.4	0.174	12.0	0.5	12.7	0.7	0.418	12.4	0.5	11.5	0.9	0.392
**TOVA VARZT**	Season 1 pre	-2.3	0.3	-3.5	1.1	0.300	-2.5	0.3	-2.0	0.6	0.417	-2.0	0.3	-3.0	0.5	0.099	-2.5	0.3	-2.1	0.7	0.647
	Season 1 post	-1.9	0.2	-3.4	0.9	0.083	-2.1	0.2	-1.4	0.4	0.147	-1.9	0.3	-2.3	0.4	0.326	-2.1	0.2	-1.8	0.4	0.488
	Season 2 pre	-2.1	0.3	-2.5	0.5	0.541	-2.2	0.3	-1.8	0.6	0.590	-2.1	0.3	-2.3	0.4	0.578	-2.3	0.3	-1.6	0.3	0.146
	Season 2 post	-1.8	0.3	-3.4	0.5	0.007	-2.1	0.3	-0.8	0.3	0.002	-2.0	0.3	-1.9	0.5	0.822	-2.0	0.3	-2.2	0.7	0.805
	Season 3 pre	-1.7	0.4	-2.8	0.9	0.308	-1.9	0.4	-1.5	0.1	0.386	-1.6	0.5	-2.3	0.5	0.266	-1.9	0.4	-1.2	0.3	0.190
	Season 3 post	-1.9	0.2	-2.5	1.1	0.605	-2.0	0.3	-1.4	0.7	0.418	-1.7	0.2	-2.5	0.5	0.141	-1.9	0.3	-1.9	0.3	0.903
	Season 4 pre	-1.6	0.2	-3.8	2.6	0.381	-1.9	0.4	-1.4	0.4	0.387	-1.5	0.3	-2.5	1.0	0.294	-1.8	0.4	-1.8	0.1	0.944
	Season 4 post	-2.2	0.3	-1.1	0.0	0.002	-2.1	0.3	-1.7	0.4	0.341	-2.3	0.4	-1.5	0.5	0.184	-1.9	0.3	-3.0	0.7	0.154

© 2021 Rose SC et al. *JAMA Network Open.*

		ADHD					Anxiety/Depression					Headache/Migraine					Previous Concussion(s)				
		No		Yes		P-value	No		Yes		P-value	No		Yes		P-value	No		Yes		P-value
		Mean	SE	Mean	SE		Mean	SE	Mean	SE		Mean	SE	Mean	SE		Mean	SE	Mean	SE	
**CogState Processing Speed**	Season 1 pre	104.8	1.4	98.6	2.1	0.016	104.6	1.3	96.4	3.6	0.035	103.4	1.8	104.8	1.7	0.578	103.7	1.4	107.1	3.1	0.303
	Season 1 post	103.1	1.5	87.3	4.5	0.001	101.2	1.7	99.8	1.8	0.572	99.6	2.2	103.4	1.9	0.191	100.6	1.7	105.0	3.3	0.234
	Season 2 pre	94.3	2.2	88.3	5.6	0.323	93.4	2.2	94.0	3.6	0.904	96.0	2.6	89.5	3.3	0.118	93.3	2.2	94.6	5.8	0.831
	Season 2 post	97.5	1.7	83.6	3.6	0.000	95.5	1.9	98.5	0.9	0.155	94.6	2.1	97.8	2.9	0.366	95.2	1.8	99.7	4.7	0.365
	Season 3 pre	92.8	1.8	83.2	3.9	0.024	92.0	1.8	89.2	5.3	0.622	92.9	1.9	89.5	3.3	0.376	91.7	1.8	91.8	4.7	0.970
	Season 3 post	90.3	2.4	79.8	3.0	0.006	89.5	2.4	84.0	3.1	0.163	89.4	2.9	88.1	3.1	0.746	88.5	2.5	91.7	4.1	0.497
	Season 4 pre	93.1	2.7	89.1	7.0	0.598	92.6	2.8	92.9	3.3	0.943	93.9	3.1	90.1	4.2	0.456	92.4	2.9	94.1	2.1	0.634
	Season 4 post	95.1	2.9	59.3	6.5	0.000	94.1	3.4	69.7	0.8	0.000	93.0	3.9	88.9	7.0	0.616	91.8	3.9	92.0	7.1	0.980
**SWAN Total**	Season 1 pre	1.9	0.3	5.6	1.3	0.005	2.4	0.3	1.6	0.8	0.356	2.2	0.5	2.8	0.8	0.581	2.3	0.3	2.9	1.1	0.624
	Season 1 post	1.6	0.3	4.1	0.9	0.012	2.0	0.3	1.6	0.6	0.544	1.8	0.5	2.3	0.7	0.576	2.0	0.3	1.8	0.5	0.638
	Season 2 pre	1.8	0.3	7.2	1.4	0.000	2.5	0.4	2.0	0.6	0.488	2.6	0.6	2.8	0.9	0.915	2.6	0.4	1.5	0.8	0.222
	Season 2 post	1.5	0.3	3.6	1.0	0.044	1.7	0.3	2.2	0.8	0.589	1.9	0.6	2.0	0.6	0.924	1.7	0.4	2.0	0.7	0.713
	Season 3 pre	2.0	0.6	10.8	1.0	0.000	3.0	0.8	3.5	1.5	0.764	2.8	0.8	3.6	1.3	0.603	3.3	0.8	1.6	0.6	0.089
	Season 3 post	1.4	0.4	6.0	0.5	0.000	1.8	0.4	3.0	0.9	0.259	2.0	0.5	1.9	0.7	0.923	1.9	0.4	2.2	1.0	0.805
	Season 4 pre	0.9	0.3	6.7	1.0	0.000	1.2	0.4	4.0	0.9	0.007	1.5	0.6	1.6	0.6	0.948	1.4	0.5	2.0	0.7	0.515
	Season 4 post	1.2	0.4	6.0	2.4	0.052	1.3	0.5	5.0	2.2	0.095	2.3	0.8	0.9	0.6	0.160	1.5	0.6	3.0	1.3	0.287
**SDQ Total Difficulties**	Season 1 pre	6.3	0.5	8.2	1.2	0.125	6.1	0.5	11.6	1.3	0.000	6.1	0.6	7.1	0.7	0.251	6.5	0.5	6.9	1.6	0.822
	Season 1 post	5.7	0.6	7.4	1.3	0.229	5.5	0.6	10.8	1.6	0.002	5.9	0.8	6.0	0.7	0.913	6.0	0.6	5.6	1.6	0.842
	Season 2 pre	5.0	0.5	10.0	2.5	0.050	5.3	0.6	9.8	1.7	0.012	5.0	0.7	6.7	1.1	0.211	5.6	0.7	6.5	1.6	0.585
	Season 2 post	4.1	0.6	8.4	1.5	0.008	4.1	0.6	9.3	1.1	0.000	4.0	0.7	5.8	1.0	0.145	4.5	0.6	5.6	1.8	0.564
	Season 3 pre	4.3	0.7	10.8	2.8	0.027	4.1	0.6	12.5	1.8	0.000	3.9	0.8	7.3	1.4	0.037	4.7	0.8	7.4	2.3	0.266
	Season 3 post	3.8	0.7	6.0	1.6	0.218	3.6	0.6	7.0	2.7	0.218	4.1	0.7	3.8	1.2	0.812	3.5	0.5	6.8	2.6	0.223
	Season 4 pre	2.9	0.5	11.0	1.6	0.000	3.2	0.6	8.3	2.7	0.063	3.7	0.8	4.0	1.5	0.840	3.4	0.7	5.8	2.1	0.304
	Season 4 post	3.4	0.7	6.0	2.4	0.306	3.3	0.7	6.3	2.6	0.266	4.5	0.9	2.1	1.0	0.074	3.3	0.7	6.0	2.3	0.249

© 2021 Rose SC et al. *JAMA Network Open.*

		ADHD					Anxiety/Depression					Headache/Migraine					Previous Concussion(s)				
		No		Yes		P-value	No		Yes		P-value	No		Yes		P-value	No		Yes		P-value
		Mean	SE	Mean	SE		Mean	SE	Mean	SE		Mean	SE	Mean	SE		Mean	SE	Mean	SE	
**SCAT-3 Total**	Season 1 pre	2.9	0.5	2.7	0.6	0.801	2.4	0.4	7.8	3.1	0.085	2.7	0.5	3.1	0.8	0.628	2.6	0.4	4.6	1.7	0.264
	Season 1 post	3.0	0.8	3.3	1.2	0.830	2.3	0.5	12.6	5.0	0.041	2.0	0.6	4.6	1.4	0.090	2.8	0.8	5.0	1.2	0.133
	Season 2 pre	2.5	0.5	4.0	1.9	0.434	2.3	0.5	6.3	1.9	0.051	1.7	0.5	4.1	1.0	0.027	2.4	0.5	4.5	1.4	0.157
	Season 2 post	2.6	0.7	5.5	3.4	0.403	2.3	0.7	8.8	3.3	0.055	2.5	0.9	3.9	1.5	0.409	2.7	0.9	4.8	1.5	0.238
	Season 3 pre	2.5	1.1	2.8	1.6	0.887	1.6	0.6	9.5	5.4	0.143	1.4	0.6	4.5	2.2	0.181	1.2	0.4	10.0	4.4	0.046
	Season 3 post	2.3	0.9	3.3	1.5	0.580	1.5	0.4	9.3	4.8	0.105	1.8	0.6	3.5	1.9	0.406	1.4	0.4	8.0	4.0	0.106
	Season 4 pre	1.1	0.6	6.3	3.6	0.151	1.2	0.6	5.7	3.8	0.248	2.2	1.1	0.8	0.3	0.222	1.0	0.6	5.5	2.8	0.120
	Season 4 post	1.8	0.6	2.0	0.9	0.843	1.6	0.5	3.7	1.7	0.225	2.2	0.6	1.0	0.7	0.208	1.3	0.4	4.5	1.8	0.089

ADHD: attention deficit hyperactivity disorder, SE: standard error, WASI FSIQ 2: Wechsler Abbreviated Scale of Intelligence 2nd Edition Full Scale intelligence quotient two subtests, WISC: Wechsler Intelligence Scale for Children 5th Edition, ChAMP: Child and Adolescent Memory Profile, TMT: Trail Making Test, TOVA: Test of Variables of Attention, VARZT: Response Time Variability z-score, SWAN: Strengths and Weakness of ADHD Symptoms and Normal Behavior Rating Scale, SDQ: Strengths and Difficulties Questionnaire, SCAT-3: Sport Concussion Assessment Tool 3rd Edition symptom score.

© 2021 Rose SC et al. *JAMA Network Open.*
